# Macrophage–*Neisseria gonorrhoeae* Interactions: A Better Understanding of Pathogen Mechanisms of Immunomodulation

**DOI:** 10.3389/fimmu.2018.03044

**Published:** 2018-12-21

**Authors:** Alejandro Escobar, Paula I. Rodas, Claudio Acuña-Castillo

**Affiliations:** ^1^Laboratorio Biología Celular y Molecular, Instituto de Investigación en Ciencias Odontológicas, Facultad de Odontología, Universidad de Chile, Santiago, Chile; ^2^Laboratorio de Microbiología Médica y Patogénesis, Facultad de Medicina, Universidad Andrés Bello, Concepción, Chile; ^3^Departamento de Biología, Facultad de Química y Biología, Universidad de Santiago de Chile, Santiago, Chile

**Keywords:** innate immunity, macrophages, *Neisseria gonorrhoeae*, host response, immunomodulation

## Abstract

*Neisseria gonorrhoeae* is a significant health problem worldwide due to multi-drug resistance issues and absence of an effective vaccine. Patients infected with *N. gonorrhoeae* have not shown a better immune response in successive infections. This might be explained by the fact that *N. gonorrhoeae* possesses several mechanisms to evade the innate and adaptative immune responses at different levels. Macrophages are a key cellular component in the innate immune response against microorganisms. The current information suggests that gonococcus can hijack the host response by mechanisms that involve the control of macrophages activity. In this mini review, we intend to condense the recent knowledge on the macrophage–*N. gonorrhoeae* interactions with a focus on strategies developed by gonococcus to evade or to exploit immune response to establish a successful infection. Finally, we discuss the opportunities and challenges of therapeutics for controlling immune manipulation by *N. gonorrhoeae*.

## Introduction

*Neisseria gonorrhoeae* or gonococcus, a gram-negative diplococcus, belongs to the genus *Neisseria* and is the etiological agent of the sexually transmitted bacterial infection (STI) gonorrhea. Nowadays, gonorrhea is the second most common bacterial STI and results in substantial morbidity and economic cost worldwide. The majority of infections are benign mucosal infections of the urogenital tract, pharynx, and rectum. Ascended infections such as endometritis, salpingitis, epididymitis, and pelvic inflammatory disease (PID) are more difficult to treat. Gonococcal PID and its related complications (infertility, ectopic pregnancy, and chronic pelvic pain) constitute the major morbidity and mortality associated with gonorrhea (1). The most recent annual incidence estimates, based on data collected in 2012, indicate 78 million new cases worldwide with a global incidence rate of 19 per 1,000 females and 24 per 1,000 males (2, 3). Another serious concern is the current identification of antimicrobial drug resistance of *N. gonorrhoeae*, which includes the most recent fluoroquinolones and extended-spectrum cephalosporin (4–6). Indeed, U.S. Centers for Disease Control (CDC) identified multidrug resistance as among the 3 most “urgent” hazard-level threats to the U.S. population (7).

Recent information shows that *N. gonorrhoeae* can prevent the development of a successful protective immune response; The causes of the weak immune response triggered by *N. gonorrhoeae* are varied and correspond to multiple mechanisms that include the immune privilege of the reproductive tissue colonized by bacteria (8, 9), as well as the own strategies developed by the bacteria, such as epitope mimicry, antigenic variations and phase variation. Moreover, gonococci seem to directly interfere with the cells involved in adaptive immune response, such as dendritic cells and B and T lymphocytes (10–13). Regarding innate immunity, phagocytic cells such as macrophages and neutrophils (PMN) represent the earliest line of defense against invading bacteria. However, PMN cannot clear infections by *N. gonorrhoeae* (14). Since gonococcus can survive into PMN and suppress the oxidative burst (14, 15), it has been hypothesized that bacteria actively recruit PMN to permit the spread to profounder tissues of the host and even to other hosts.

Moreover, *N. gonorrhoeae* also affects macrophages and their functionality, posing additional difficulties to its detection and elimination by the innate immune system (16).

Considering that macrophages are critical cells in the innate immune response to *N. gonorrhoeae*, and macrophage-driven innate immune response can be subverted by *N. gonorrhoeae*, promoting the persistence of gonorrhea, we will highlight our current knowledge about macrophage–*N. gonorrhoeae* interactions in this mini review with a focus on strategies developed by gonococcus to evade or to exploit immune response to establish a successful infection.

## Macrophages and Antimicrobial Response

Macrophages are present in almost all tissues and have diverse functions ranged from clearance of microbes, dead, and senescent cells until reparative and regulatory functions. Tissue-resident macrophages can derive from yolk sac macrophages, fetal liver monocytes, or adult bone-marrow monocytes capable of entering in tissues during inflammation (17, 18). When bacteria cross the layer of epithelial cells, accessing submucosa, they have the first encounter with macrophages (19). Macrophages recognize microbial pathogen-associated molecular patterns (PAMPs) and endogenous danger-associated molecular patterns (DAMPs) by genome-encoded pattern recognition receptors (PRRs). It leads the activation of host defense pathways, which include internalization of the pathogen into phagosomes and fusion with lysosomes to form phagolysosomes, where the microbes are killed by reactive oxygen and nitrogen species and proteolytic enzymes, resulting in the clearance of the infection (20). Also, activation of macrophages includes secretion of pro-inflammatory cytokines and antigen-presentation (21). Currently, it has been demonstrated that many signals recognized by macrophages drive to classical activation (M1 macrophages) or alternative activation (M2 macrophages) (22). While M1 macrophages stimulate a robust anti-tumor and microbicide response, M2 macrophages are involved in tumor progression, tissue remodeling, response against parasites and they have regulatory functions (23).

## Cellular Model for Examining Macrophages During Infection

Diverse cell models have been used to study the interaction between *N. gonorrhoeae* and macrophages; these include cell lines and primary macrophages. The most common murine line used is RAW 264.7. This is a tumor line induced by Abelson murine leukemia virus (24). Human macrophage cell lines mostly used include U937 and THP-1. THP-1 cells are derived from acute monocytic leukemia (25) and U-937 cells were obtained from a patient suffering from histiocytic lymphoma (26). We should consider the differences between these lines based on their diverse origin and maturation stage (27).

Moreover, cell lines generally vary from primary macrophages, since repeated subculture typically results in some abnormalities such as the loss of genes expression (28); although this is not important for proliferation, it is critical for the immune function developed by macrophages. Because of these deficiencies of macrophage cell lines, it may be essential to complement such a model with culture of primary macrophages cells. Cell sources for primary macrophage models include mouse peritoneal macrophages (PM), bone marrow-derived macrophages (BMDM) and human monocyte-derived macrophage (MDM). Murine source of macrophages can be obtained from different strains of wild-type, transgenic and gene-targeted mice. Although PM can be easily harvested, the output is small and we should consider that sanitary and welfare conditions of the animal could affect the physiology of macrophages (29).

Conversely, BMDM do not present problems associated with the health status of the donor mice, because BMDM are obtained from bone marrow stem cells and are completely differentiated *in vitro* (29). In this line, human peripheral blood monocytes are the most commonly used precursors for generating macrophages *in vitro*. The MDM culture allows the monocytes differentiation and polarization toward different macrophage phenotypes using cytokines and/or bacterial products. Thus, M1 and M2 macrophages require GM-CSF or M-CSF in the presence of IFN-γ and/or LPS and IL-4 or IL-13, respectively (30, 31).

## Mechanisms of Survival and Replication of *N. gonorrhoeae* During Macrophage Infection

*Neisseria gonorrhoeae* is able to prevent some defense mechanisms elicited by macrophages. Our laboratory and others have showed different mechanisms used by gonococcus to evade antimicrobial macrophage response. Below, we discuss some of the most relevant mechanisms observed in Macrophage–*N. gonorrhoeae* interactions.

## Modulation of Phagocytosis by Macrophage

*Neisseria gonorrhoeae* can avoid phagocytosis mediated by opsonic antibodies. The membrane molecules such as lipooligosaccharide (LOS), Pili, Opa, and Porin (Por) are the main targets to generate antibodies against gonococci (32–34). By constantly varying these antigens, the bacteria elude antibody opsonization and successive IgG Fc-receptor-mediated phagocytosis (14, 35, 36). Additionally, LOS carbohydrates moiety mimic human surface antigens (37, 38) impairing the recognition by macrophages. Other membrane components such as pili reduce the association *in vitro* between *N. gonorrhoeae* and non-activated mouse PM macrophages, similar to streptococcal M protein which reduce the phagocytosis (39). However, the rate of phagocytosis of piliated gonococcus can be enhanced using immune anti-serum, indicating that this enhancement reflects primarily immunoglobulin G-mediated phagocytosis (opsonization through Fc receptor) rather than surface attachment (40, 41). In spite of this, later studies showed that pili (particularly pilin subunit PilE) acts as a factor that promotes non-opsonic ingestion of gonococci by human monocytes in absence of serum factors rather than as a protective factor (42).

## Modulation of Intracellular Killing by Macrophage

It has been demonstrated that human monocytes and macrophages can kill intracellular gonococci (39) with diverse kinetics, ranged from a complete killing after 30 min of incubation in mouse peritoneal macrophages (43) to a more prolonged period of survival in RAW 264.7 cells and human MDM (44). When using membrane-impermeable antibiotics, such as gentamicin, our group and others have seen that the number of intracellular gonococcus inside macrophages increases over time (27, 45, 46). The persistence of *N. gonorrhoeae* inside macrophages is indicative of resistance mechanisms to innate host defenses. Thus, studies in monocytes showed that Opa-positive and piliated gonococci caused a differential oxidative response (42). Château et al. showed in differentiated U937 cells that internalized bacteria could escape from phagosome or endosome and retain viability (27).

Moreover, studies using MDM showed that purified PorB modifies phagosomal processing and reduces the delivery of the lysosomal enzyme cathepsin D, suggesting a delay in phagosome maturation and oxidative killing mechanisms (47), which is consistent with the absence of colocalization of *N. gonorrhoeae* with Lysosomal-associated membrane protein 1(LAMP-1) present in the lysosomal acidic compartment (27). In this way, two other proteins have been described, Ng-MIP (with homology to Macrophage infectivity potentiator), and Ng-OmpA (with homology to the outer membrane protein A). Ng-OmpA, unlike Ng-MIP, has been associated with the adhesion and internalization of *N. gonorrhoeae* in RAW 264.7 cells (44, 48). However, both proteins have shown that they protect bacteria from being killed by macrophages, probably through mechanisms related to the activity of peptidylprolyl cis/trans isomerase (PPIase) (44) and the inhibition of apoptosis of infected macrophages (48), as observed in other pathogens (49–51). Intracellular survival is also favored by the modulation of cellular iron metabolism by gonococcus. Infection of human monocytes, THP-1, MM6, and murine RAW 264.7 cell lines showed an upregulation of hepcidin, neutrophil gelatinase-associated lipocalin (NGAL) and Natural resistance-associated macrophage protein 1 (NRAMP1), suggesting an increase in cellular iron bioavailability (52). Similarly, infection of BMDM with *N. gonorrhoeae* was associated with increased availability of intracellular iron, associated with INF-β and cGAS/STING signaling pathway (53).

## Modulation of Macrophage Cell Death

Apoptosis and autophagy constitute cell death modes for the elimination of infected cells. Failure in these mechanisms can favor the advance of disease (54, 55). *Neisseria gonorrhoeae* infection and its effects on apoptosis have been studied in PMN, and epithelial cells showed different results (56–59). A study using various sources of macrophages showed that gonococcus inhibit apoptosis in U937 and MDM cells in intrinsic and extrinsic pathways, but in THP-1 cells only extrinsic apoptotic pathway was affected (27). Concerning autophagy, Zughaier et al. showed that a phosphoethanolamine (PEA) modification of lipid A increases *N. gonorrhoeae* survival through evasion of autophagy affecting the TLR4-mediated induction of autophagic flux in RAW264 and THP-1 macrophages (60). Additionally, cell death can arise by pyroptosis. Studies using *N. gonorrhoeae* or isolated *N. gonorrhoeae* LOS showed cell death through NLRP3-mediated pyronecrosis in THP-1 cells (61) and cell death in human macrophages by activation of both canonical and non-canonical pyroptosis pathways (62).

## Modulation of Macrophage Activation

Several membrane components from *N. gonorrhoeae* including LOS play a role during infection and modulation of antimicrobial response (63, 64). The immunomodulatory potential of LOS is based on lipid A backbone rather than carbohydrates structure. In this context, Patrone et al. showed that the variation in carbohydrates structure from LOS has no influence on production of IL-8, IL-12, and TNF-α cytokines (65). Indeed, THP-1 cells showed differences in their inflammatory response, TLR4/MD2 signaling or TNF-α production, toward LOS derived from a variety of *Neisseria* strains independent of the oligosaccharide truncation (66). Contrarily, the presence of single lauric acid residue in a determinate position of Lipid A and a hexa-acylated lipid A seems to be critical for the initiation of pro-inflammatory responses, since *lpxLII* and *msbB* mutants that lost these features showed a reduced ability to induce pro-inflammatory cytokines and stimulation of TLR-4-inflammatory signaling in U937, THP-1 and mouse primary macrophages (67, 68).

Similarly, modification of gonococcal lipid A with PEA also influences the production of CXCL3 and CXCL10 chemokines in infected macrophages (60). Besides, Knilans et al. showed that other molecules like peptidoglycan monomers released during *N. gonorrhoeae* cell wall remodeling by LtgA and LtgD lytic transglycosylases, also suppress TNF-α and IL-1β production in THP-1 cells by modulation TLR-2 and NOD2 signaling pathways (69). In this way, it is possible to hypothesize that Opa binding to CEACAM1 present on monocytes may suppress the activation of this cell, similarly to CD4 T cells (70, 71).

## Modulation of Macrophage Phenotype

Macrophages are part of reproductive tissues together with epithelial and stromal cells both in humans and mice (72). Considering that reproductive organs, which are targets of *N. gonorrhoeae* infection, have a privileged immune status (73–75), one can speculate that macrophages could contribute to regulatory response. Findings of our laboratory have demonstrated that gonococcus affects murine RAW264 and its functionality. We showed a shift toward the production of IL-10 and TGF-β and inefficient up-regulation in molecules involved in antigen presentation such as MHC class II and CD86, and therefore a weak allogeneic T-cell stimulatory activity (45).

Similarly, differentiated macrophages from THP-1 and U937 cells challenged with *N. gonorrhoeae* showed induction of IL-10 and low levels of IL-12 (27). In this line, we have also demonstrated that MDM challenged with *N. gonorrhoeae* were differentiated toward an M2 profile, expressing the CD163 marker, inducing anti-inflammatory cytokines and inhibitory surface molecules with a low capacity to stimulate CD4 T cells proliferation (46). Regarding this, it is crucial to implement studies that characterize the phenotype of macrophages in a murine model or the genital tract of the patient.

## Opportunities and Challenges of Therapeutics to Control Immune Manipulation

Due to multi-drug resistance problems related to gonorrhea, researchers and clinicians are dedicated to making preventive vaccines. However, the capacity to create an effective vaccine has not been possible yet due to the characteristics of the immune response against *N. gonorrhoeae* (76). Today, the development of gonorrhea vaccines has not been effective. Only one study has shown reduced rates of gonorrhea infection in patients immunized with outer membrane vesicle meningococcal B vaccine (MeNZB) with an effectiveness of 31% (77). However, these findings are the first evidence in humans lately. Thus, other types of treatments become necessary. Liu et al. assessed an intravaginal treatment using IL-12–loaded microspheres in a murine model of infection. Microencapsulated IL-12 transforms the infection in a “live vaccine” that triggers the production of local and systemic gonococcus-specific antibodies (78). This method was also evaluated together with a non-living gonococcal vaccine based on gonococcal outer membrane vesicles (OMV) showing an elicitation of long-term humoral protective immunity driven by a Th1 response, which was effective against antigenically diverse strains of *N. gonorrhoeae* (79). Besides, the blockade of TGF-β1 and IL-10 has been useful to reverse *N. gonorrhoeae*-mediated suppression of Th1 and Th2 responses and it facilitates the development of specific protective immunity (11, 80). Moreover, Youssef et al. hypothesized the use of curcumin and vitamin D to “rescue” the immunity, by counteracting the different ways used by *N. gonorrhoeae* to evade the immune response (76). Considering that gonococcus affects macrophages polarization toward M2 phenotype (42), a putative treatment which would reverse M2 to M1 polarization is likely to be beneficial, since M1 polarization is associated with control of acute infections by many intracellular bacteria (81). In this regard, Na et al. blocked M-CSF-induced M2 macrophages differentiation using a COX-2 inhibitor Etodolac driving pro-inflammatory activities in human and murine macrophages and proposing it as a therapy for induction of enhanced anti-tumor immunity (82). In this line, we think that the M2 phenotype promoted by gonococcus could be reverted using COX-2 inhibitor. We also showed no significant differences in IL-1β levels in MDM infected cells compared to non-infected MDM cells, suggesting that *N. gonorrhoeae* could trigger insufficient IL-1β levels to activate innate immune response (83). Then, we were able to promote the IL-1β processing and release using exogenous ATP. Interestingly, production of IL-1β did not correlate with the activation of inflammasome-mediated caspase-1, suggesting that ATP could be acting at the level of mechanisms related to vesicle trafficking or pore formation.

We also showed a significant up-regulation of co-inhibitory molecule Programmed Death Ligand 1 (PD-L1) and CD86 down-regulation in macrophages upon gonococcus infection (46). Considering that in the immunological synapse the outcome of the immune response relies at least on balance between positive (CD86) and negative (PD-L1) co-stimulation signals, we hypothesize that an increase in PD-L1 expression, particularly in macrophages, represents a strategy used by *N. gonorrhoeae* to inhibit previously activated T cells at the later stages of the immune response in peripheral tissues. It could be reversed by PD-L1 blockade, as it has been shown for the treatment of severe chronic infectious diseases such as LCMV, HIV, HCV, HBV, C*. trachomatis*, and *T. crassiceps* infection (84–87).

## Conclusion

**Figure 1:**
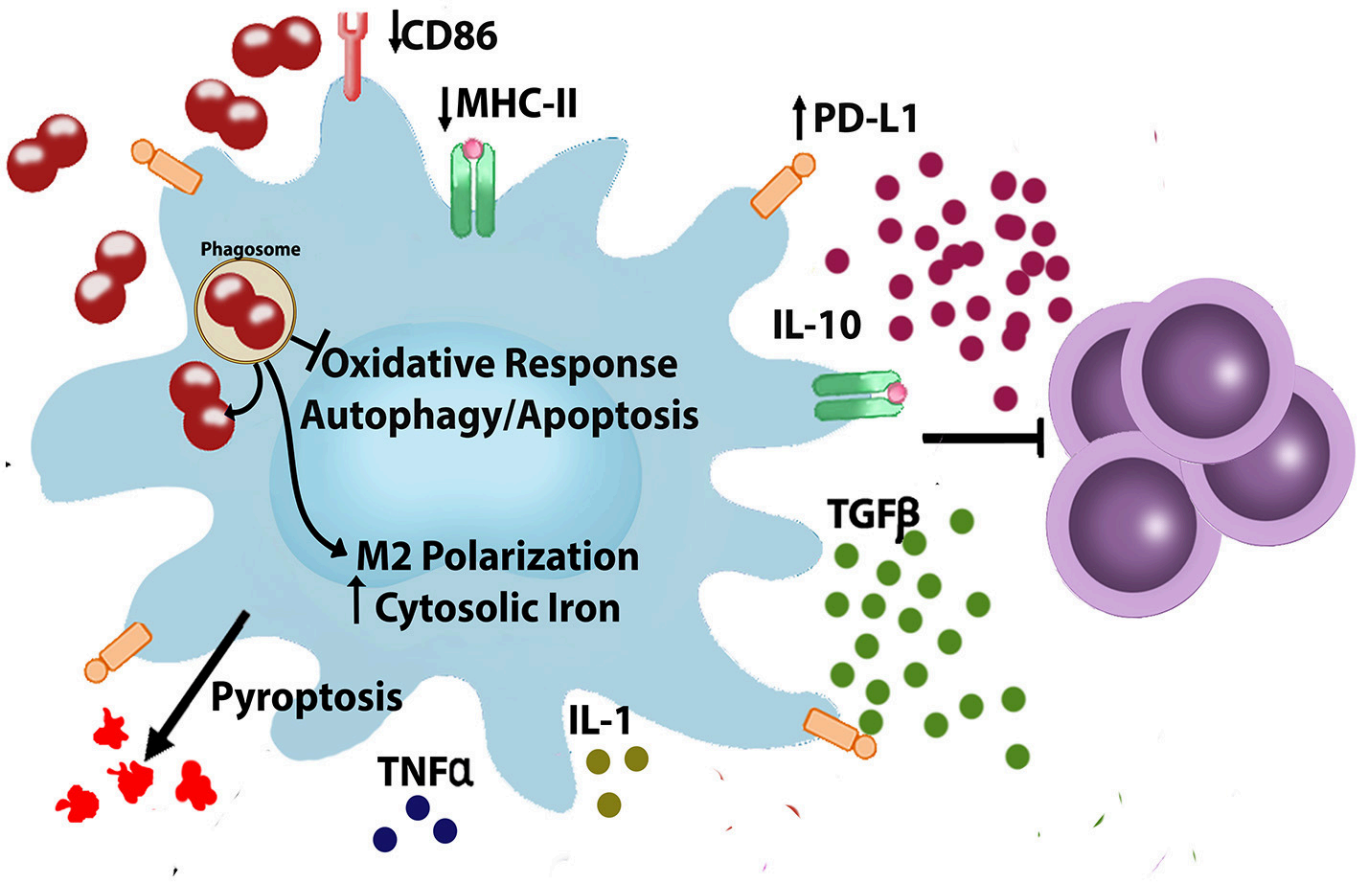
*Neisseria gonorrhoeae* evades and modulates macrophages. During infection, *N. gonorrhoeae* interact with immune cells such as macrophages. In macrophages *N. gonorrhoeae* is able to escape from phagosome (27, 42, 45–48), modulate cellular iron metabolism (52, 53), inhibit apoptosis and autophagy (27, 60), modulate production of inflammatory/anti-inflammatory cytokines (63–71, 83), and polarizes macrophages, resulting in macrophages that are less capable of T cell proliferation (45). CD86, Cluster of Differentiation 86; IL- 1, Interleukin 1; IL-10, Interleukin 10; MHC, Major Histocompatibility Complex; PD-L1, Programmed Death-Ligand 1; TGF-β, Transforming Growth Factor-beta; TNF-α, Tumor Necrosis Factor-alpha.

Until now, much of the research associated with modulation of innate immune cell response has been focused on gonococcus-PMN interaction, and many advances in this field have been achieved. Nevertheless, the interaction of *N. gonorrhoeae* with the resident or recruited macrophages is less well understood. Our current knowledge of the *N. gonorrhoeae* interactions with macrophages shows the capacity of gonococcus to avoid the response to an infectious challenge (phagocytosis and effective killing) and the elicitation of acquired immune response (Figure 1). Although we are still investigating which are the molecules from *N. gonorrhoeae* that confer resistance to macrophage, we have advanced in many questions about modulation of macrophage response and its role in gonococcal pathogenesis. There are still questions associated with the signaling pathways and cellular mechanisms related to inflammatory response, as well as the effects in the T cell activation and differentiation. Accessibility to mouse and human macrophages and the capacity of genetic or pharmacological manipulation will facilitate the studies addressed to find potential targets to be exploited with new therapeutic approaches beyond antimicrobial drugs.

## Author Contributions

AE conceived and designed the mini review and wrote the mini review. PR contributed to the writing and critically revised the paper. CA-C critically revised the paper. All authors read and approved the submitted version.

### Conflict of Interest Statement

The authors declare that the research was conducted in the absence of any commercial or financial relationships that could be construed as a potential conflict of interest.
